# Functional Maps of Protein Complexes from Quantitative Genetic Interaction Data

**DOI:** 10.1371/journal.pcbi.1000065

**Published:** 2008-04-18

**Authors:** Sourav Bandyopadhyay, Ryan Kelley, Nevan J. Krogan, Trey Ideker

**Affiliations:** 1 Program in Bioinformatics, University of California San Diego, La Jolla, California, United States of America; 2 Department of Bioengineering, University of California San Diego, La Jolla, California, United States of America; 3 Department of Cellular and Molecular Pharmacology, University of California San Francisco, San Francisco, California, United States of America; Keck Graduate Institute of Applied Life Sciences, United States of America

## Abstract

Recently, a number of advanced screening technologies have allowed for the comprehensive quantification of aggravating and alleviating genetic interactions among gene pairs. In parallel, TAP-MS studies (tandem affinity purification followed by mass spectroscopy) have been successful at identifying physical protein interactions that can indicate proteins participating in the same molecular complex. Here, we propose a method for the joint learning of protein complexes and their functional relationships by integration of quantitative genetic interactions and TAP-MS data. Using 3 independent benchmark datasets, we demonstrate that this method is >50% more accurate at identifying functionally related protein pairs than previous approaches. Application to genes involved in yeast chromosome organization identifies a functional map of 91 multimeric complexes, a number of which are novel or have been substantially expanded by addition of new subunits. Interestingly, we find that complexes that are enriched for aggravating genetic interactions (i.e., synthetic lethality) are more likely to contain essential genes, linking each of these interactions to an underlying mechanism. These results demonstrate the importance of both large-scale genetic and physical interaction data in mapping pathway architecture and function.

## Author Summary

Biologists are currently producing large amounts of data focused on physical and genetic protein interactions. Physical interactions dictate the architecture of the cell in terms of how direct associations between molecules constitute protein complexes, while genetic interactions define functional relationships through cause-and-effect relationships between genes. Both of these types of interactions can indicate shared protein functions; however, these two types of interactions are commonly non-overlapping, making their interpretation difficult. Along these lines, it has been noted that genetic interactions commonly occur between members of the same protein complex as well as between functionally related complexes. Here, we present an integrated framework that incorporates both types of interactions to generate large maps of protein complexes as well as highlight connections between related complexes. The ability to rapidly integrate these two types of data in an automated fashion can accelerate the discovery of new members of protein complexes as well as identify functionally related cellular components.

## Introduction

Genetic interactions are logical relationships between genes that occur when mutating two or more genes in combination produces an unexpected phenotype [1]–[3]. Recently, rapid screening of genetic interactions has become feasible using Synthetic Genetic Arrays (SGA) or diploid Synthetic Lethality Analysis by Microarray (dSLAM) [4],[5]. SGA pairs a gene deletion of interest against a deletion to every other gene in the genome (in turn). The growth/no growth phenotype measured over all pairings defines a *genetic interaction profile* for that gene, with no growth indicating a synthetic-lethal genetic interaction. Alternatively, all combinations of double deletions can be analyzed among a functionally-related group of genes [6]–[8]. A recent variant of SGA termed E-MAP [9] has made it possible to measure continuous rates of growth with varying degrees of epistasis (based on imaging of colony sizes). “Aggravating” interactions are indicated if the growth rate of the double gene deletion is slower than expected, while for “alleviating” interactions the opposite is true [10],[11].

One popular method to analyze genetic interaction data has been to hierarchically cluster genes using the distance between their genetic interaction profiles. Clusters of genes with similar profiles are manually searched to identify the known pathways and complexes they contain as well as any genetic interactions between these complexes. This approach has been applied to several large-scale genetic interaction screens in yeast including genes involved in the secretory pathway [8] and chromosome organization [6]. Segré et al. [12] extended basic hierarchical clustering with the concept of “monochromaticity”, in which genes were merged into the same cluster based on minimizing the number of interactions with other clusters that do not share the same classification (aggravating or alleviating).

Another set of methods has sought to interpret genetic relationships using physical protein-protein interactions [13]. Among these, Kelley and Ideker [14] used physical interactions to identify both “within-module” and “between-module” explanations for genetic interactions. In both cases, modules were detected as clusters of proteins that physically interact with each other more often than expected by chance. The “within-module” model predicts that these clusters directly overlap with clusters of genetic interactions. The “between-module” model predicts that genetic interactions run between two physical clusters that are functionally related. This approach was improved by Ulitsky *et al.* [15] using a relaxed definition of physical modules. In related work, Zhang et al. [16] screened known complexes annotated by the Munich Information Center for Protein Sequences (MIPS) to identify pairs of complexes with dense genetic interactions between them.

One concern with the above approaches, and the works by Kelley and Ulitsky in particular, is that they make assumptions about the density of interactions within and between modules which have not been justified biologically. Ideally, such parameters should be learned directly from the data. Second, between-module relationships are identified by separate, independent searches of the network seeded from each genetic interaction. This “local” search strategy can lead to a set of modules that are highly overlapping or even completely redundant with one another. Finally, genetic interactions are assumed to be binary growth/no growth events while E-MAP technology has now made it possible to measure continuous values of genetic interaction with varying degrees of epistasis. Here, we present a new approach for integrating quantitative genetic and physical interaction data which addresses several of these shortcomings. Interactions are analyzed to infer a set of modules and a set of inter-module links, in which a module represents a protein complex with a coherent cellular function, and inter-module links capture functional relationships between modules which can vary quantitatively in strength and sign. Our approach is supervised, in that the appropriate pattern of physical and genetic interactions is not predetermined but learned from examples of known complexes. Rather than identify each module in independent searches, all modules are identified simultaneously within a single unified map of modules and inter-module functional relationships. We show that this method outperforms a number of alternative approaches and that, when applied to analyze a recent EMAP study of yeast chromosome function, it identifies numerous new protein complexes and protein functional relationships.

## Results

### Characterization of Genetic and Physical Networks

We first sought to quantitatively confirm whether, and to what degree, physical and genetic interactions could indicate common membership in a protein complex. To provide genetic data for analysis, we obtained the previously-published results from a large E-MAP of yeast chromosomal biology [6]. This study consisted of genetic interactions measured among 743 genes (including 74 essential genes), yielding quantitative values for 182,669 gene pairs (66% of all possible pair-wise measurements). Each gene pair was assigned an S-score, where positive scores indicate protein pairs for which the double mutant grows better than expected (i.e., an alleviating interaction) and negative scores indicate pairs for which the double mutant grows worse than expected (i.e., a synthetic sick/lethal or aggravating interaction) where the expectation is that the double-deletion of unrelated proteins will have a growth rate equivalent to the multiplicative product of the two individual growth rates [17]. In all, 14,237 gene pairs (8%) showed strong genetic interactions with |S|>2.5. Physical interactions were taken from a recent computational integration of two large datasets measured by co-immunoprecipitation followed by mass spectrometry [18]. This study assigned to each pairwise interaction a Purification Enrichment (PE) score, with larger values representing a greater likelihood of true binding.

**Figure 1:**
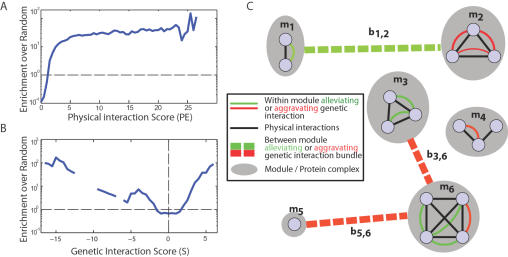
Combining physical and genetic interactions to define protein complexes. Correspondence of the physical interaction score (A) and the genetic interaction score (B) with the known small-scale, manually annotated protein complexes in MIPS. To compute the enrichment over random (*y*-axis), one first computes the fraction *f* of interactions at each score *x* that fall inside a MIPS small-scale complex (bin size of 1.5). The enrichment is the ratio *f*/*r*, where *r* is the fraction of random protein pairs within MIPS complexes. (C) Proteins are grouped into physically interacting sets called modules (gray ovals; *m*_1_–*m*_6_). Pairs of modules may be linked to indicate a functional relationship (dotted lines; *b*_1_–*b*_6_). The assignment of proteins to modules along with the list of inter-module links comprises the state of the system.

Figure 1A confirms that protein pairs with higher PE-scores are more likely to operate in a known small-scale protein complex recorded in the MIPS database [19] (versus protein pairs chosen at random). This result is expected considering that PE-scores were trained based on these complexes [18]. Figure 1B shows that protein pairs with both positive and negative S-scores are more likely to operate within a known complex. Positive (alleviating) interactions are well-known to occur between subunits of a complex [6]. Negative (aggravating) interactions are to a lesser degree so, although the mechanism has not been as clear as for the alleviating case [20]. By comparing the magnitudes of enrichment between Figures 1A and 1B, it is apparent that extreme S-scores are at least as indicative of co-complex membership as strong PE-scores, if not more so (∼100-fold enrichment versus ∼50-fold enrichment, respectively). Together, these exploratory findings suggest that the physical and genetic information can indeed provide a basis for the identification of protein pairs involved in the same complex.

### Functional Maps of Protein Complexes Involved in Yeast Chromosomal Biology

To capture these trends, we formulated an approach to classify a protein pair as operating either within the same module or between functionally related modules given its genetic and physical interaction scores. This approach seeks to categorize interactions supported by both strong genetic and physical evidence as operating within a module (i.e., complex). Interactions with a strong genetic but weak physical signal are better characterized as operating between two functionally related modules. Given within-module and between-module likelihoods for individual interactions, an agglomerative clustering procedure seeks to merge these interactions into increasingly larger modules and to identify pairs of modules interconnected by bundles of many strong genetic interactions (Figure 1C). Full details are provided in Methods.

**Figure 2:**
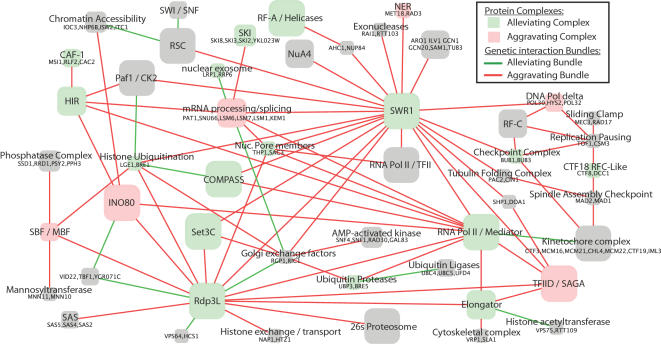
Global map of protein complexes involved in yeast chromosome biology. Each node represents a predicted multimeric protein complex, while each link represents a significantly alleviating or aggravating bundle of genetic interactions between complexes, indicative of an inter-complex functional relationship. Node colors indicate enrichment for alleviating or aggravating genetic interactions among members of the same complex. Node sizes are proportional to the number of proteins in the complex. When known, nodes are labeled with the common name of the complex. For complexes that are newly identified by our study and thus unnamed, the constituent proteins are listed. For clarity, the co-chaperone prefoldin complex (PFD1, PAC10, YKE2, GIM3, GIM4, GIM5, BUD27) and the 25 links associated with it have been removed.

Applying this method, we identified 91 distinct modules with an average size of 4.1 proteins per module. Figure 2 gives an overview of a subset of the identified modules and inter-module links. Complete results are catalogued at http://www.cellcircuits.org/Bandyopadhyay2008/html/. Overall, these results suggest ten novel complexes not recorded in either the small-scale or high-throughput MIPS compendium, covering 23 proteins in total. The results also identify 84 new subunits of known complexes (Dataset S1). Through permutation testing, 19 versus 9 of the identified modules could be categorized as enriched for alleviating or aggravating genetic interactions, respectively. A total of 313 significant genetic relationships were identified between modules, 94 versus 219 of which were enriched for alleviating or aggravating interactions.

### Comparison to Related Approaches

**Figure 3:**
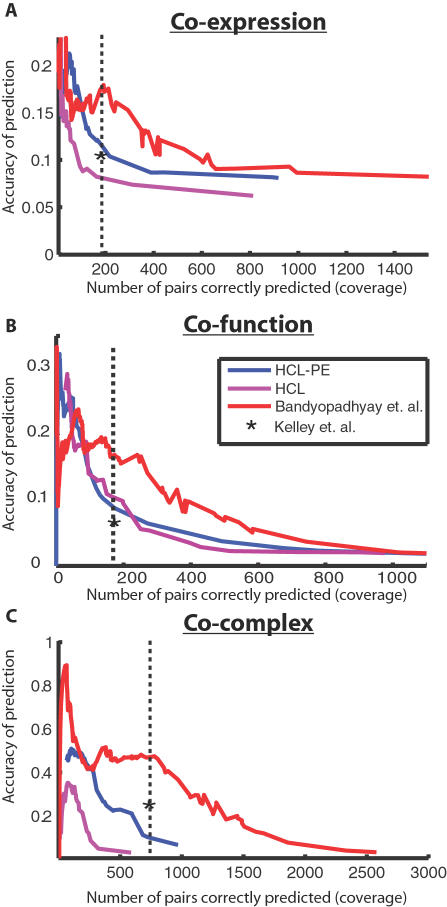
Performance of complex identification. The proposed approach is compared to several competing methods of discovering protein complexes within genetic interaction networks: HCL implements hierarchical clustering with a distance measure computed from the genetic interaction profiles only (S-scores), while HCL-PE extends HCL by merging clusters only if there is a physical interaction between them (PE-score>1). For the modules defined by each method, accuracy versus coverage is plotted over a range of values for tuning the module size (see Methods). Accuracy is estimated as the fraction of protein pairs in a predicted module that are in a gold-standard set; coverage is estimated as the number of gold-standard pairs that fall in the same predicted module. Gold-standard sets are defined by protein pairs that are either (A) co-expressed, (B) functionally-related, or (C) assigned to the same complex in high-throughput data sets (as annotated in MIPS). The performance at the chosen parameter setting (*α* = 1.6) is indicated by the dotted vertical line. The performance of the method of Kelley *et al.* is reported for the same level of coverage as the present approach (asterisk). Since it operates on binary interaction data, we converted quantitative genetic and physical interaction scores to binary values based on a threshold of |S|>2.5 and PE>1.

The method of choice for interpreting quantitative genetic interactions has been hierarchical clustering (HCL) of genes based on pair-wise distances between their genetic interaction profiles [6],[8]. We compared the clusters obtained using HCL to the modules obtained with our present approach (Bandyopadhyay *et al.*) using three gold-standard metrics: gene co-expression (Figure 3A), co-functional annotation (Figure 3B), or membership in the same previously-identified complex (Figure 3C). To ensure a fair comparison between the two approaches, HCL and Bandyopadhyay *et al.* were evaluated across a range of coverages (number of gold-standard gene pairs recovered by the predicted clusters/modules; see Methods). For all three benchmarks, our performance was substantially higher than that of the HCL-based approach at most levels of coverage (and at a level of coverage corresponding to the 91 modules reported above; dotted vertical line in Figure 3).

We considered that one reason why HCL performed less favorably might be that it was not given access to the same information (i.e., the physical network). This is especially true for the metric based on previously identified complexes, in which complexes were annotated based on the same high-throughput protein interactions used here. To investigate this possibility, we extended HCL to incorporate physical interactions in a straightforward fashion, by merging only those clusters which share a physical interaction between them (HCL-PE). Although this approach outperformed hierarchical clustering without physical interactions, it was outperformed by the present approach by at least 50% across the three metrics. Finally, our method also shows improvement over the previous approach of Kelley and Ideker [14] for integrating genetic and physical interactions (Figure 3).

### Aggravating Complexes Tend to be Essential

Nineteen versus nine of the learned modules had significant enrichment for alleviating versus aggravating genetic interactions, respectively. Identification of “alleviating” modules is expected, since subunits of a complex operate together and the phenotypic effect of removing any pair of proteins in a complex should be no worse than removing any single protein individually. The presence of aggravating interactions within modules was more intriguing. One way in which aggravating interactions could occur among the subunits of a complex is if its function is essential, i.e., the loss of the complex's function causes a lethal phenotype. In these cases, some protein subunits should be encoded by essential genes, while other subunits might be redundant and thus essential in pairwise combinations [20].

**Figure 4:**
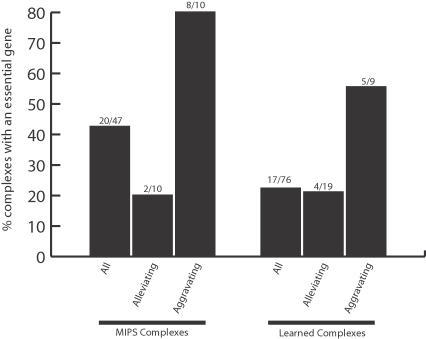
Aggravating complexes are more likely to contain essential genes. The percentage of complexes that contain at least one essential gene is shown, for various groups of complexes defined within small-scale complexes in MIPS (left three bars) or complexes identified in this study (right three bars). In MIPS, approximately 80% of “aggravating” complexes (see text) contain an essential gene, versus 20% for “alleviating” complexes. The trend is similar for the complexes reported in this study, with 55% versus 22% of aggravating versus alleviating complexes containing an essential gene. The list of all essential genes was taken from (http://www-sequence.stanford.edu/group/yeast_deletion_project/deletions3.html).

To test the hypothesis that essential genes are more likely in aggravating modules, we analyzed both MIPS small-scale complexes and our learned modules for the presence of essential genes (Figure 4). We found that 80% of aggravating MIPS complexes contained an essential gene, compared to only 20% of alleviating MIPS complexes (a four-fold increase). Similarly, of the aggravating modules determined by our approach, 55% contained an essential gene compared to only 21% of alleviating modules (a 2.6-fold increase). These results are not correlated with module size, as the median size of aggravating learned modules is less than the median size of alleviating learned modules. They suggest that, regardless of the technique for identifying complexes, those containing essential genes tend to be composed of primarily aggravating genetic interactions. Mechanistically, this might occur through a variety of means, including proteins with separate but functionally redundant roles in maintaining complex integrity, or subunit substitution by paralogous proteins.

## Discussion

**Figure 5:**
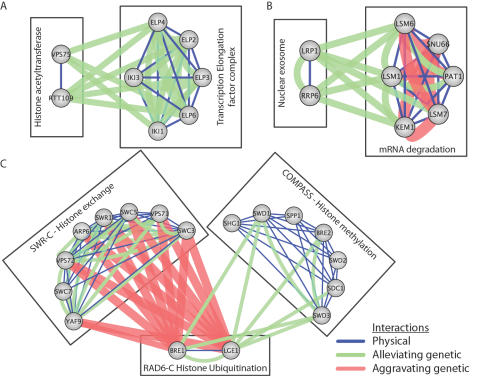
Pathway models identify novel functional associations among cellular machinery. Each panel represents complexes and between-complex links taken from Figure 2. Physical interactions with PE>1 are shown and strong genetic interactions (|S|>2.5) are shown with increased thicknesses corresponding to stronger genetic interactions. (A) Histone acetyltransferase complex RTT109 – VPS75 showing strong alleviating interactions with the Elongator transcription elongation factor complex. (B) Between-complex model highlighting alleviating interactions between the LRP1 – RRP6 nuclear exosome complex and an mRNA degradation complex. (C) Complexes associated with the RAD6-C histone ubiquitination complex (BRE1/LGE1).

Figure 5 presents detailed diagrams of example functional relationships elucidated by our module mapping method. Figure 5A shows the alleviating relationship between the RTT109-VPS75 histone acetyltransferase complex [6],[21],[22] and Elongator, a complex that is associated with RNA Polymerase II and is involved in transcriptional elongation [23]. Since several subunits both of Elongator and RTT109/VPS75 have been shown to be involved in histone acetylation levels [22],[24], these two complexes may operate together to effectively clear histones from actively transcribed regions. To identify further mechanisms of their cooperation, future studies may search for specific residues of histone H3 whose acetylation levels are modulated by both complexes. This example highlights the utility of an integrated approach, since although RTT109 and VPS75 are known to form a complex their genetic interaction profiles are not congruent (correlation of profiles of −0.1) and had been missed by hierarchical clustering. Figure 5B highlights non-essential components (LRP1 and RRP6) of the exosome, which contributes to the quality-control system that retains and degrades aberrant mRNAs in the nucleus [25]. These components have alleviating interactions with a complex composed of Lsm proteins involved in mRNA decay.

Figure 5C centers on BRE1/LGE1, subunits of the Rad6 Histone Ubiquitination Complex (RAD6-C; the Rad6 protein itself was not covered by the original E-MAP screen) [26],[27]. RAD6-C is functionally connected with two other complexes, SWR-C and COMPASS. SWR-C functions to regulate gene expression through the incorporation of transcriptionally active histone variant H2AZ [28]–[30], while COMPASS is involved in mediating transcriptional elongation and silencing at telomeres through methylation of histone H3 [31]. Interactions between RAD6-C and SWR are aggravating, suggesting synergy or redundancy towards an essential cellular function. Interactions between RAD6-C and COMPASS are alleviating, suggesting they operate in a potentially serial fashion. Consistent with this analysis, it has been shown that histone H2B ubiquitination by RAD6-C is a prerequisite for histone H3 methylation by COMPASS [32],[33].

Several trends emerge from the performance analysis in Figure 3. First, genetic interaction data alone can yield substantial information about molecular pathways. Functionally similar proteins often have similar profiles of genetic interaction, a feature we have previously exploited to identify functional interactions between complexes as well as to identify new members of complexes based on a combination of weak physical and genetic data [14]. On the other hand, the ability to detect complexes can be greatly improved by adding information about protein physical interactions. Even the straightforward HCL-PE method was able to greatly improve the accuracy and coverage according to most metrics, while the greatest performance was achieved by the improved probabilistic framework we have presented in this study. This framework has led to the inclusion of YKL023W as a potential new member of the SKI complex and YGR071C in a complex with VID22/TBF1 (Figure 2), for a total of 84 novel protein subunit assignments to complexes (Dataset S1). Both of these examples have both physical and genetic support and would have been missed by an approach based on either type of interaction alone.

Future work may seek to incorporate yet additional types of linkages such as protein-DNA interactions [34],[35], kinase-substrate phosphorylations [36], or other genetic perturbation data such as eQTLs [37]. There are also opportunities to refine the modeling framework further. Here, a gold-standard set of complexes was used to explicitly learn the relationship between physical interactions, genetic interactions, and module membership. This supervised approach could be extended to also learn which features best indicate the inter-module functional relationships, perhaps through curation of a gold-standard set of interacting complexes.

## Methods

### Problem Definition

We analyze the interaction data to infer *a set of protein modules* and *a set of inter-module links* (Figure 1C). A protein module is defined as a set of proteins that are connected through protein-protein interactions and are likely to represent a protein complex with a coherent cellular function. Inter-module links capture functional relationships between modules and may be of two types, aggravating or alleviating. The complete state of the system is described by a set *M* of modules, each module defining a set of proteins, and a set *N* of pairs of modules that are functionally linked.

### Scoring Module Co-Membership

For each pair of proteins (*a*,*b*) we compute a log ratio *W* of the likelihood that *a* and *b* fall *within* the same module versus the likelihood that they are unrelated (i.e., occur in the background). The function uses two sources of information that are indicative of protein complex co-membership: the strength of protein-protein physical interaction (*PE*) and the strength of genetic interaction (*S*):
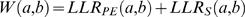
(1)For a given data type (*PE* or *S*) the log likelihood ratio (LLR) is defined as:
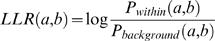
(2)The probability *P_within_* is determined using logistic regression training on 217 complexes curated from small-scale studies in MIPS [19]. *P_background_* is the probability of randomly observing the observed value (*PE* or *S*) for the pair (*a*,*b*) in the background of all gene pairs. As shown in Figure 1A and 1B, it is clear that higher values of *PE* are predictive of MIPS complex membership. As both positive and negative values of *S* are predictive, *LLR_S_*(*a,b*) is trained on the absolute value of *S*. A third predictor based on the correlation of genetic interaction profiles was also evaluated but did not result in any gain in performance (Figure S1).

### Scoring Inter-Module Links

A similar function *B*() is formulated to assess the likelihood that two proteins fall *between* modules that are functionally linked. The function inputs the same two sources of information on protein-protein and genetic interactions (*PE* and *S*). Unfortunately, there is no curated set of functionally related complexes that can be used as positive training examples for regression. Instead, *B*() is derived from the within-module LLRs, assuming that between-module interactions have a similar pattern of genetic interactions but lack physical interactions:
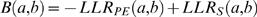
(3)This function captures both aggravating and alleviating genetic interactions between two functionally-related modules. It also ensures such modules are physically separate—if not, they would be better considered as a single module.

### Global Optimization of Module Memberships and Links

Given the above functions *W*() and *B*(), we compute the likelihood of the complete system (i.e., given a particular choice *M* of modules and *N* of inter-module links):
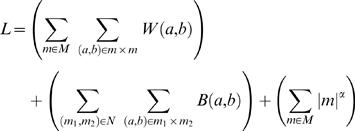
(4)The first term accumulates the within-module scores among gene pairs assigned to the same module. The second term accumulates the inter-module scores for gene pairs spanning any two modules. Gene pairs spanning unlinked modules do not contribute to *L*. The final term is a tunable reward which scales with module size. Larger values of *α* result in fewer, larger complexes. The final module map shown in Figure 2 was generated using *α* = 1.6, based on its good coverage and performance across all three metrics in Figure 3.

### Module Search

Assignment of gene to modules and of inter-module links is performed using a simple variant of UPGMA hierarchical clustering [38]: (a) Initially, each gene is assigned to a separate module; (b) Each pair of modules (*m_1_*, *m_2_*) is evaluated for merging into a single module *m* = *m*_1_∪*m*_2_; the pair-wise merging that results in the largest increase in *L* is chosen; (c) Repeat step b until no module merge operation increases *L*.

At each iteration of step b, *L* is optimized over all possible ways of assigning inter-module links (i.e., module pairs are linked whenever the second term in Equation 4 is positive). Because each inter-module link is scored independently, additions or deletions of links from the system need only be evaluated for modules that are under evaluation for merging.

Subsequent to the above procedure, each between-module link is evaluated to assess its significance and whether it represents predominantly aggravating or alleviating genetic interactions. A two-tailed p-value is computed by indexing the sum of *S*-scores for gene pairs falling across the two modules against a distribution of 10^6^ sums of equal numbers of *S*-scores drawn from random gene pairs. To account for multiple testing, we use the distribution of between-module p-values to compute a local false discovery rate (FDR) [39]. All reported between-module links have an inferred FDR of <10% with the global map in Figure 2 constrained to links with an FDR of <1%. Module maps in Figure 2 and Figure 5 are visualized using the Cytoscape package [40],[41].

To label modules as “aggravating” or “alleviating” (Figure 2), the sum of *S*-scores for gene pairs assigned to the same module is compared to a distribution of sums of equal numbers of randomly drawn *S*-scores. Modules with a two-tailed p-value<0.05 are labeled as either alleviating (right tail) or aggravating (left tail).

### Validation Using Co-Expression, Co-Function, or Co-Complex Annotations

Co-expressed gene pairs were defined using gene expression datasets culled from the Stanford Microarray Database covering ∼790 conditions [42]. The validation set was taken as the top 5% (13,014) of pairs ranked by Pearson correlation coefficient. The co-function set was based on yeast Gene Ontology annotations from November 2005 which predates the publication of large scale TAP-MS studies that were used to generate the PE-score [43]. This set was taken as the top 5% (13,052) most functionally similar gene pairs covered in the E-MAP. Functional similarity was determined by comparison to the background probability of picking two genes with the same shared functional annotation from the entire yeast genome (via a hypergeometric test). Similar analysis using current Gene Ontology annotation was also performed **(** Figure S2). The co-complex validation set was defined as gene pairs from 846 MIPS complexes annotated using high-throughput approaches (with interactions also appearing in small-scale studies removed) for a total of 2,885 gold-standard pairs.

The size and number of final modules was varied by altering the *α* parameter (see above). To assess performance at low coverage we ran the method with no reward contribution (remove the third term in Equation 4 by setting α = −∞) and plotted the performance of the algorithm at each merge step, which ultimately connects with the performance of the method as *α* is increased. For HCL and HCL-PE methods, the size and number of modules were varied by changing the level at which the hierarchy was cut.

## Supporting Information

Figure S1 Addition of congruence as a predictor of pathway membership.
(0.10 MB DOC)

Figure S2 A current version of the Gene Ontology shows similar performance.
(0.09 MB DOC)

Dataset S1 Results tables in Excel format.
(0.06 MB XLS)
