# Supplementary material for: Functional Maps of Protein Complexes from Quantitative Genetic Interaction Data
Source: PLoS Comput Biol. 2008 Apr 18;4(4):e1000065. doi: 10.1371/journal.pcbi.1000065 (PMC2289880; doi:10.1371/journal.pcbi.1000065)
Supplement: Figure S1 — Addition of congruence as a predictor of pathway membership. (0.10 MB DOC) [file pcbi.1000065.s001.doc]

**
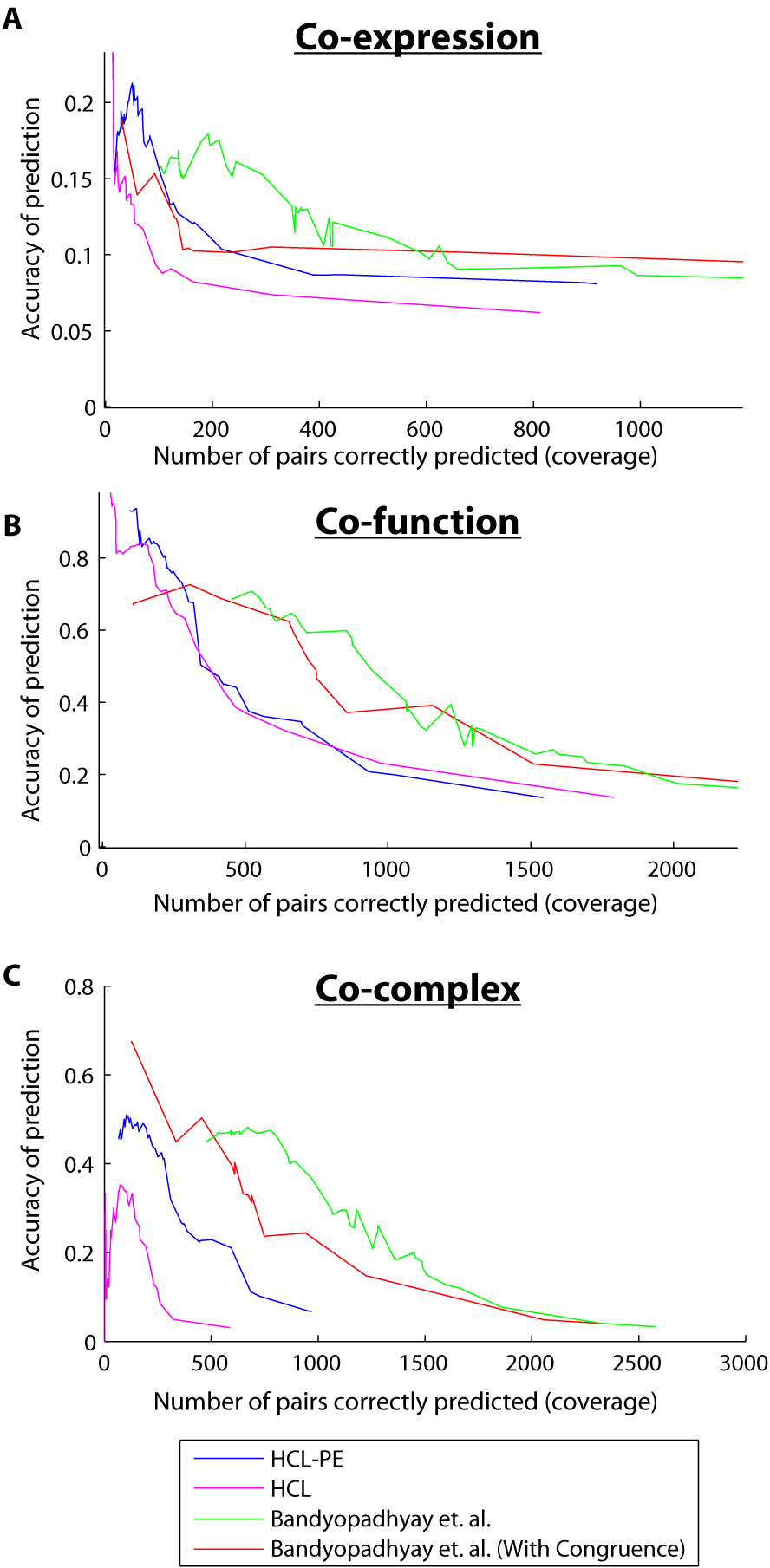
**

**Figure S1: Addition of congruence as a predictor of pathway membership.** A variant of this algorithm which includes congruence (measured as the pearson correlation of genetic interaction profiles) was included as a third predictor (beyond pairwise physical and genetic interaction scores)**.** The results indicate that, especially in determining co-complex membership, the addition of congruence does not help to find functionally related modules. A possible rationale for this result is that by scoring between-complex interactions explicitly, the method is already rewarding for similarity of genetic interaction profiles so that the addition of the third congruence predictor results in overfitting and no additional gain in performance.
