# Supplementary material for: Functional Maps of Protein Complexes from Quantitative Genetic Interaction Data
Source: PLoS Comput Biol. 2008 Apr 18;4(4):e1000065. doi: 10.1371/journal.pcbi.1000065 (PMC2289880; doi:10.1371/journal.pcbi.1000065)
Supplement: Figure S2 — A current version of the Gene Ontology shows similar performance. (0.09 MB DOC) [file pcbi.1000065.s002.doc]

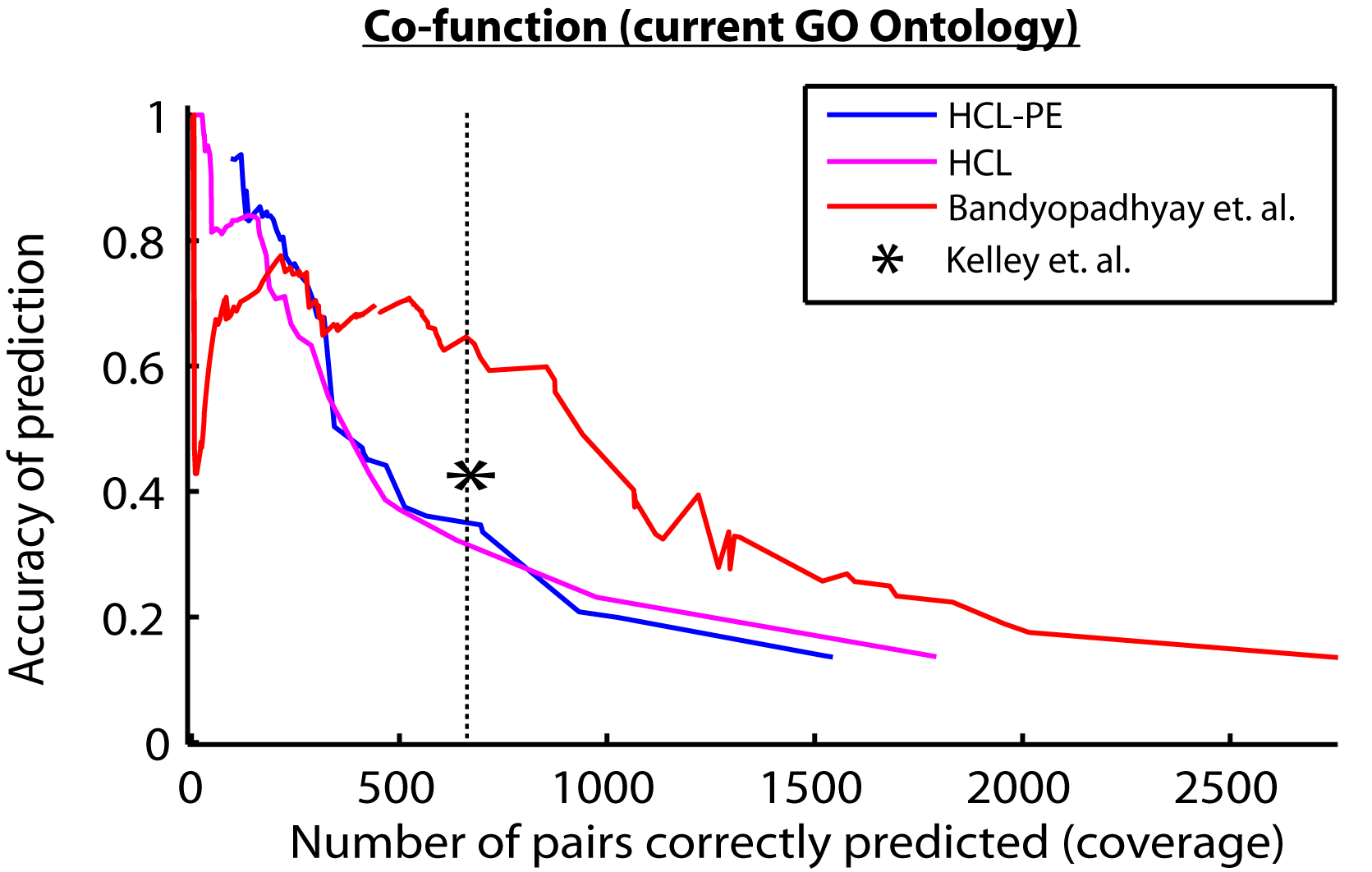


**Figure S2: A current version of the Gene Ontology shows similar performance.** The figure is the same as Figure 3B using the current version of the Gene Ontology (March 2007).
